# Long non‐coding RNA expressed in macrophage co‐varies with the inflammatory phenotype during macrophage development and polarization

**DOI:** 10.1111/jcmm.14557

**Published:** 2019-08-16

**Authors:** Yixin Xie, Min Wang, Jingjing Tian, Xianping Li, Min Yang, Kan Zhang, Shan Tan, Lingli Luo, Can Luo, Longkai Peng, Aiguo Tang

**Affiliations:** ^1^ Department of Laboratory Medicine, The Second Xiangya Hospital Central South University Changsha Hunan China; ^2^ Department of Urological Organ Transplantation, Center of Organ Transplantation, The Second Xiangya Hospital Central South University Changsha Hunan China

**Keywords:** cancer, immune response, inflammation, long non‐coding RNA, macrophage polarization

## Abstract

Advances in microarray, RNA‐seq and omics techniques, thousands of long non‐coding RNAs (lncRNAs) with unknown functions have been discovered. LncRNAs have presented a diverse perspective on gene regulation in diverse biological processes, especially in human immune response. Macrophages participate in the whole phase of immune inflammatory response. They are able to shape their phenotype and arouse extensive functional activation after receiving physiological and pathological stimuli. Emerging studies indicated that lncRNAs participated in the gene regulatory network during complex biological processes of macrophage, including macrophage‐induced inflammatory responses. Here, we reviewed the existing knowledges of lncRNAs in the processes of macrophage development and polarization, and their roles in several different inflammatory diseases. Specifically, we focused on how lncRNAs function in macrophage, which might help to discover some potential therapeutic targets and diagnostic biomarkers.

## 1 INTRODUCTION

Macrophage plays a crucial intermediary role in human immune response. They can be activated after innate immunity receptors sensing the tissue damage and metabolic dysfunction, which is considered as the first line of defence for human immunity.^1^, ^2^ In response to various stimuli, macrophages have a strong functional and phenotypic plasticity,^3^ and can be polarized to the classical M1 type and the M2 type.^4^ Toll‐like receptor (TLR) ligands, macrophage receptors with collagenous structure (MARCO) and interferon γ (IFN‐γ) may induce M1 macrophages and then promote the production of some pro‐inflammatory mediators including tumour necrosis factor‐α (TNF‐α), cytokines IL‐1, IL‐6 and IL‐23, inducible nitric oxide synthase (iNOS) and HLA‐DR.^5^ However, IL4/IL13 (M2a), immune complex (M2b), and the anti‐inflammatory cytokine IL‐10 or transforming growth factor‐β (M2c) can induce M2 macrophages and produce IL‐10, mammalian chitinase Ym1, arginase 1 (Arg1), resistin‐like molecule α1 (Fizz1), CD163 and chitotriosidase against inflammation.^6^, ^7^, ^8^ The signalling pathways involved in macrophage activation follow the common four pathways, including JNK, JAK/STAT, Notch and PI3K/Akt pathways.^8^, ^9^ Furthermore, macrophages can experience phenotypic conversion to adapt to some specific environmental changes,^10^ such as cancer,^11^ fatty liver,^12^ tissue repair and remodelling.^13^

Next‐generation RNA sequencing and omics techniques have displayed that most of the human genomes were transcribed into RNAs, and nearly 98% of them without coding for proteins. These 98% of RNAs are called non‐coding RNAs (ncRNAs).^14^ LncRNAs are ncRNAs with critical biological roles and a length of more than 200 nucleotides.^15^, ^16^, ^17^ The mechanisms of lncRNAs remain unclear,^18^ but increasing number of in vitro and in vivo studies have shown that lncRNAs can be expressed in macrophage during its development and control some gene expression.^19^, ^20^, ^21^ However, a systematic review uncovered the interactions among lncRNAs expressed in macrophage is not available. Hence, we reviewed recent advances in elucidation of the variability of lncRNA function within macrophages, and mainly focused on the following four aspects: biology of lncRNAs, involvement of lncRNAs in human monocyte/macrophage differentiation, characteristics of lncRNAs in macrophage polarization and dysregulation of lncRNAs in macrophage involved inflammatory diseases. Overall, this review may help to discover some potential therapeutic targets and diagnostic biomarkers for multiple diseases.^22^, ^23^

## 2 BIOGENESIS, STRUCTURES AND FUNCTION MANNERS OF LNCRNAS

Compared with other ncRNAs, the mechanisms of lncRNAs were still unclear because of its complexity and relatively poor conservation.^24^ Increased researches have already extensively enhanced the knowledge of lncRNAs, and some articles have processed stemmatic reviews on its biology, structures and function manners. For details, see Beermann,^14^ Fatica^25^ or Quinn.^26^ Briefly, biogenesis of lncRNAs are usually transcribed by RNA Pol II(RNPII) in the nucleus, and only a fraction are more likely to be transcribed by RNPIII.^24^ LncRNAs can be transcribed from intergenic (between protein‐coding genes, lincRNA), intronic, natural antisense transcripts (NATs) or promoters and enhancers.^27^ Bio‐functional activities of lncRNAs rely on base pairing upon the primary structure, or develop by higher‐order configurations on the basis of the secondary structures.^28^, ^29^ Diversity of their structures enabled lncRNAs to perform a variety of functions.^30^ LncRNAs share the same cap and polyadenylate tail structures with mRNAs, but express lower levels than mRNAs, and in generally exhibit more specific expression profiles and precise expression patterns.^31^, ^32^

**Figure 1:**
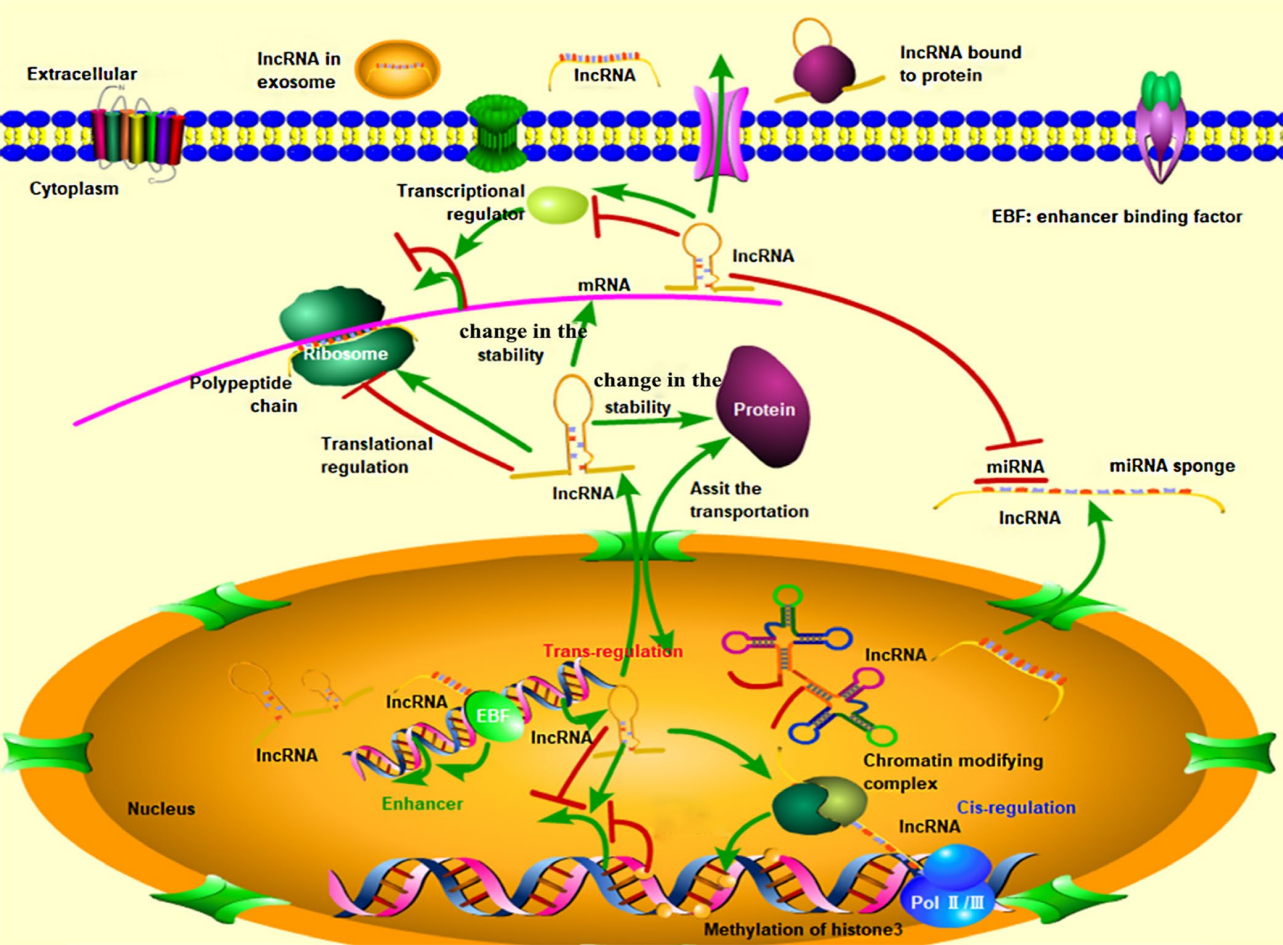
Biogenesis, structures and functions of lncRNAs. EBF: enhancer‐binding factors. The biogenesis of lncRNAs is mainly transcribed by RNPII in the nucleus. Bio‐functional activities of lncRNAs rely on base pairing upon the primary structure, or develop by higher‐order configurations on the basis of the secondary structures including helices, hairpin loops, bulges and pseudoknots. Biological functions of lncRNAs can be primarily classified into 3 types: acting as regulator of genomic transcription in the nucleus via *cis*‐ or *trans*‐acting lncRNAs and eRNAs; participating in post‐transcriptional moderation in the cytoplasm and involving in peripheral circulation.^28^, ^29^, ^30^, ^31^, ^32^, ^33^, ^34^, ^35^, ^36^, ^37^, ^38^, ^39^, ^40^, ^41^ LncRNAs promoted biological processes showed in green arrow, and negative regulated displayed in red line

The regulation of lncRNAs is also complex and diverse. Most transcribed lncRNAs tend to remain in the cell nucleus and be assembled into chromosomes during biological processes.^31^ Biological functions of most lncRNAs in accordance with previous reports can be primarily classified into 3 types^14^, ^33^: (a) LncRNAs act as regulator of genomic transcription in the nucleus. Transcription of certain genes are regulated by binding chromatin‐modifying factors, heterogeneous nuclear ribonucleoprotein (hnRNPs) or transcription factors, with *cis‐* or *trans‐*acting lncRNAs and enhancer RNAs (eRNAs). *Cis*‐regulatory way may directly silence the nearby genes of transcribed place by methylation of histone H3 and so on.^34^ However, most lncRNAs work in a *trans*‐regulatory way and play a repressor or activator role in some distant gene loci which are not even on the same chromosome.^35^ LncRNAs can also originate from enhancer elements called eRNAs, acting on the regulation of enhancer activity.^36^ (b) LncRNAs participate in post‐transcriptional moderation in the cytoplasm. These lncRNAs can promote or weaken the translation of target mRNAs, even alter the stability of mRNAs and proteins, or change the protein translocation.^29^, ^33^, ^37^, ^38^ Moreover, they can function as competing endogenous RNAs (ceRNAs), also called microRNA (miRNA) sponges, to protect the target mRNAs expression by directly binding to miRNAs or keeping miRNAs away from mRNAs.^39^ (c) The last function manner of lncRNAs is secreted to extracellular. It has been displayed that lncRNAs could be packed in extracellular vesicles (EVs), such as exosomes, secreted out of cell alone or bound to proteins.^40^ Circulating lncRNAs show advantages in biomarkers, regulating many pathophysiological processes^41^ (shown in Figure 1).

## 3 LNCRNAS INVOLVE IN HUMAN MONOCYTE/MACROPHAGE DIFFERENTIATION

**Figure 2:**
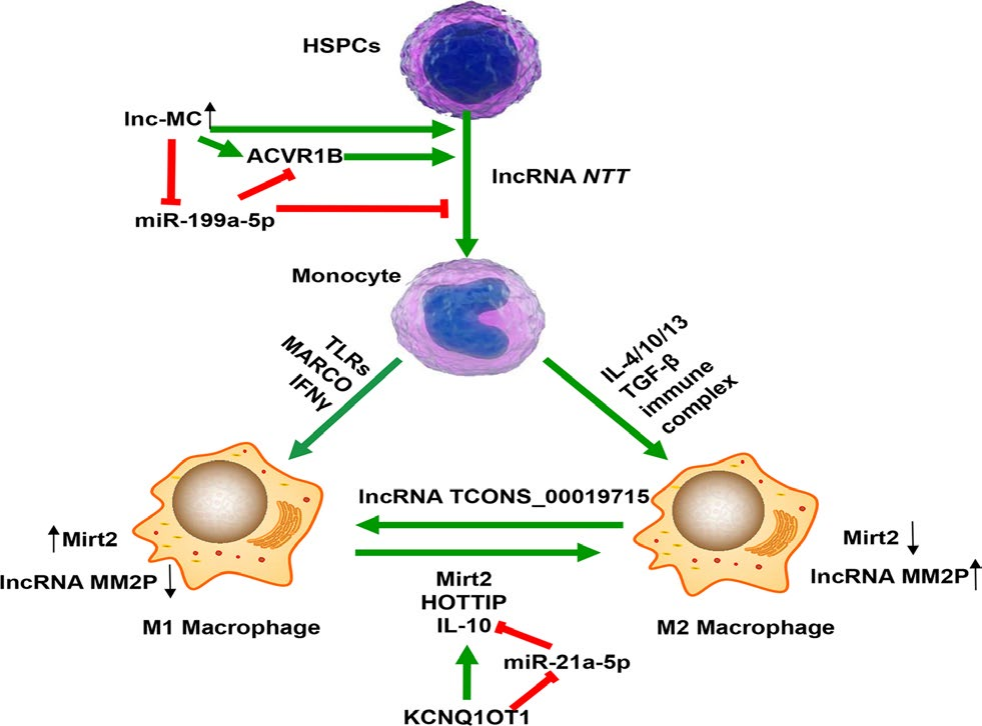
Involvement of lncRNAs in monocyte/macrophage development and M1/M2 switch. Circulating monocytes originated from haematopoietic stem/progenitor cells (HSPCs) are considered as the precursors of macrophages. Induced by phorbol‐12‐myristate‐13‐acetate (PMA), lnc‐MC up‐regulated in THP‐1, HL‐60 cells and CD34+ HSPCs. It could act as a ceRNA to soak up miR‐199a‐5p, and eventually facilitate the differentiation process through activating the transforming growth factor β (TGF‐β) signalling pathway. 45, 46 In response to various stimuli, macrophages mainly polarized into two types: classical M1 and alternative M2 type, and dynamically switched its function to adapt to the specific environmental changes. LPS induced up‐regulation of lncRNA Mirt2 in the cytoplasm, but the increase in Mirt2 was restricted by sustained and excessive activation of inflammatory responses at the late stage. Restoring Mirt2 expression in later stage promoted the IL‐4 induced M2 polarization with a remarkable increased level of M2 markers.^53^ HOTTIP expression was associated with TLR tolerance, and has been speculated to skew macrophage polarization to a “M2‐like” phenotype.^82^ KCNQ1OT1 could function as a miR‐21a‐5p decoy to up‐regulate IL‐10, induced polarization of macrophages into M2 type.^83^, ^84^ LncRNA TCONS_00019715 was expressed at a higher level when M2 macrophage was converted to M1, but decreased when M1 type was converted to M2^85^

Monocytes/macrophages originated from haematopoietic stem cells (HSCs) play a major role in non‐specific immunity and inflammatory response.^42^ Monocyte is a population of mononuclear leucocyte in circulating blood cells. It can transfer to diverse tissues and then differentiate into macrophage. This complex differentiation process requires a coordinated expression of transcription factors, cytokines and ncRNAs.^43^ Although many of well‐defined miRNAs have been recognized as key regulators involved in haematopoietic differentiation, there were few reports about lncRNAs regulating monocyte/macrophage differentiation.^44^ Chen et al have revealed that phorbol‐12‐myristate‐13‐acetate (PMA) could raise the long non‐coding monocytic RNA (lnc‐MC) expression in human THP‐1 cell line, HL‐60 cell line and CD34+ haematopoietic stem‐progenitor cells (HSPCs). Mechanically, lnc‐MC acted as a ceRNA to soak up miR‐199a‐5p and release activin A receptor type 1B (ACVR1B),^45^ then enhanced the effect of haematopoiesis‐specific transcription factor PU.1 on suppressing miR‐199a‐5p and eventually facilitated the differentiation process through activating the transforming growth factor β (TGF‐β).^46^ Once monocyte recruiting from bone marrow to peripheral blood, it can differentiate into macrophage with phenotypic variability.^47^ LncRNA *NTT* was found to be expressed in resting human primary monocyte, monocyte‐derived macrophage and the THP‐1 cell line. Yang et al have elucidated the role of lncRNA *NTT* in monocyte after knocking down it in THP‐1 cells. Briefly, the key transcription factor of monocyte C/EBPβ bound to the *NTT* promoter and regulated the *NTT* expression, which might enhance the *PBOV1* expression by interacting with hnRNPU and the promoter of *PBOV1*. In lipopolysaccharide (LPS)‐treated THP‐1 cells and peripheral blood mononuclear cells (PBMCs) of first‐diagnosed untreated early rheumatoid arthritis (RA) patients, the *C/EBPβ/NTT/PBOV1* axis was found to be hyperactivated. Increased expression of *PBOV1* resulted in cell cycle G1 arrest, differentiation into macrophages, high *IL‐10* and *CXCL10* mRNA levels, and up‐regulation of the costimulatory molecules^48^ (Figure 2 and Table 1).

**Table 1:** Main mechanisms of lncRNAs expressed in macrophages during development and polarization

Phases	Positive regulated	Mechanisms and models	Refs	Negative regulated	Mechanisms and models	Refs
Monocyte/macrophage differentiation	lnc‐MC	Act as miR‐199a‐5p sponge, releases it suppressor, THP‐1, HL‐60 cells and CD34^+^ HSPCs	45, 46	‐		
M1‐like macrophages induced by inflammatory response	lncRNA‐Nfkb2, lncRNA‐Rel	Unknown, BMDMs	57	lncRNA Lethe	Keep p65 subunit away from DNA and inhibit inflammatory genes expression. NOX2 expression and ROS production, RAW264.7cells	71
	AS‐IL1α	Recruit RNPII to the* IL‐1α* promoter, and elevate H3K9, BMDMs	58	lincRNA‐p21	Sequester p65 mRNA and attenuate the translation of p65, human Jurkat T cell and THP‐1 monocyte lines	72
	lncRNA cox‐2(PACER)	Interacte with the p50/p50 homodimeric and act in cis‐ as a decoy to activate the promoter of cox‐2, human and mouse macrophages	49, 59	lnc‐IL7R	Enhance trimethylation of H3K27, lead to transcriptional silence, THP‐1	73
	IL1β‐eRNA, IL1β‐RBT46	Act as eRNAs to promote the expression of IL1‐β and CXCL8, THP‐1	60	lincRNA THRIL	Interact with hnRNPL at the promoter of Tnfα to make sure the expression in dose control, human macrophages	74, 75
	lincRNA‐Tnfaip3	Interact with the HMGB1 protein and facilitate Hmgb1‐associated histone modification, murine macrophages	61, 62	lncRNA SeT	Attenuate the stability of Tnfα mRNA levels, murine macrophages	20
	lncRNA‐CCL2	Suppress histone deacetylase Sirtuin‐1, sepsis mice and LPS‐ stimulated macro phages	64	NKILA	Modify the phosphorylation motifs of IĸBα to block its degradation, breast cancer cell lines	77
	lncRNA FIRRE	Interact with hnRNPU and enhance stability of several inflammatory mRNAs, human macrophages and intestinal epithelial cells	65	lincRNA‐EPS	Function as a scaffold to bind hnRNPL, BMDMs	78
	lncRNA SNHG16	Act as a ceRNA to up‐regulate TLR4 by down‐regulating the miR‐15a/16 cluster, RAW264.7 cells	66	MALAT1	Interact with NF‐κB in the nucleus, keep it away from DNA and decrease inflammatory cytokines, THP‐1, RAW264.7 cells	79
	lincRNA‐Cox2	Assemble into SWI/SNF complex; affecting IκBα degradation in the cytoplasm; interact with hnRNP‐A2/B1 and hnRNP‐A/B to suppress inflammation; promote recruitment of Mi‐2/NuRD repressor complex, which led to increased H3K27 dimethylation at *Il12b* promoter, BMDMs	67, 68, 69, 70	MacORIS	A repressor of IFN‐γ signalling, human THP‐1 macrophages	81
	lncRNA MALAT1	Suppression of inflammatory responses by up‐regulating miR‐146a, murine alveolar macrophage cell line MH‐S	80			
M2‐like macrophages	lncRNA Mirt2	Inhibit TRAF6 oligomerization and ubiquitination, RAW264.7 cells	53	‐		
	HOTTIP	Unknown, endotoxin‐tolerized macrophages	50	‐		
	lncRNA KCNQ1OT1	Function as a miR‐21a‐5p decoy to up‐regulate IL‐10, PMMA‐induced BMDMs.	83, 84	‐		
M1/M2 switch	lncRNA TCONS_00019715	Decrease when M1 type is converted to M2, THP‐1, MDMs	85	PAK1	Be helpful to M1 macrophage polarization, THP‐1, MDMs	85

Abbreviations: BMDMs, bone marrow‐derived macrophages; H3K9, acetylation of histone H3 at lysine 9; HMGB1, high‐mobility group box 1 protein; hnRNPU, heterogeneous nuclear ribonucleoprotein; MDMs, monocyte‐derived macrophages; Mi‐2/NuRD, Mi‐2/nucleosome remodelling and deacetylase; NOX2, NADPH oxidase 2; PMMA, polymethyl methacrylate; ROS, reactive oxygen species; SWI/SNF, SWItch/Sucrose Non‐Fermentable; TRAF6, TNF receptor‐associated factor 6.

## 4 LNCRNAs EXPRESSION IN MACROPHAGE POLARIZATION AND FUNCTION

### 4.1 lncRNAs expression profile in M1‐like macrophage

**Figure 3:**
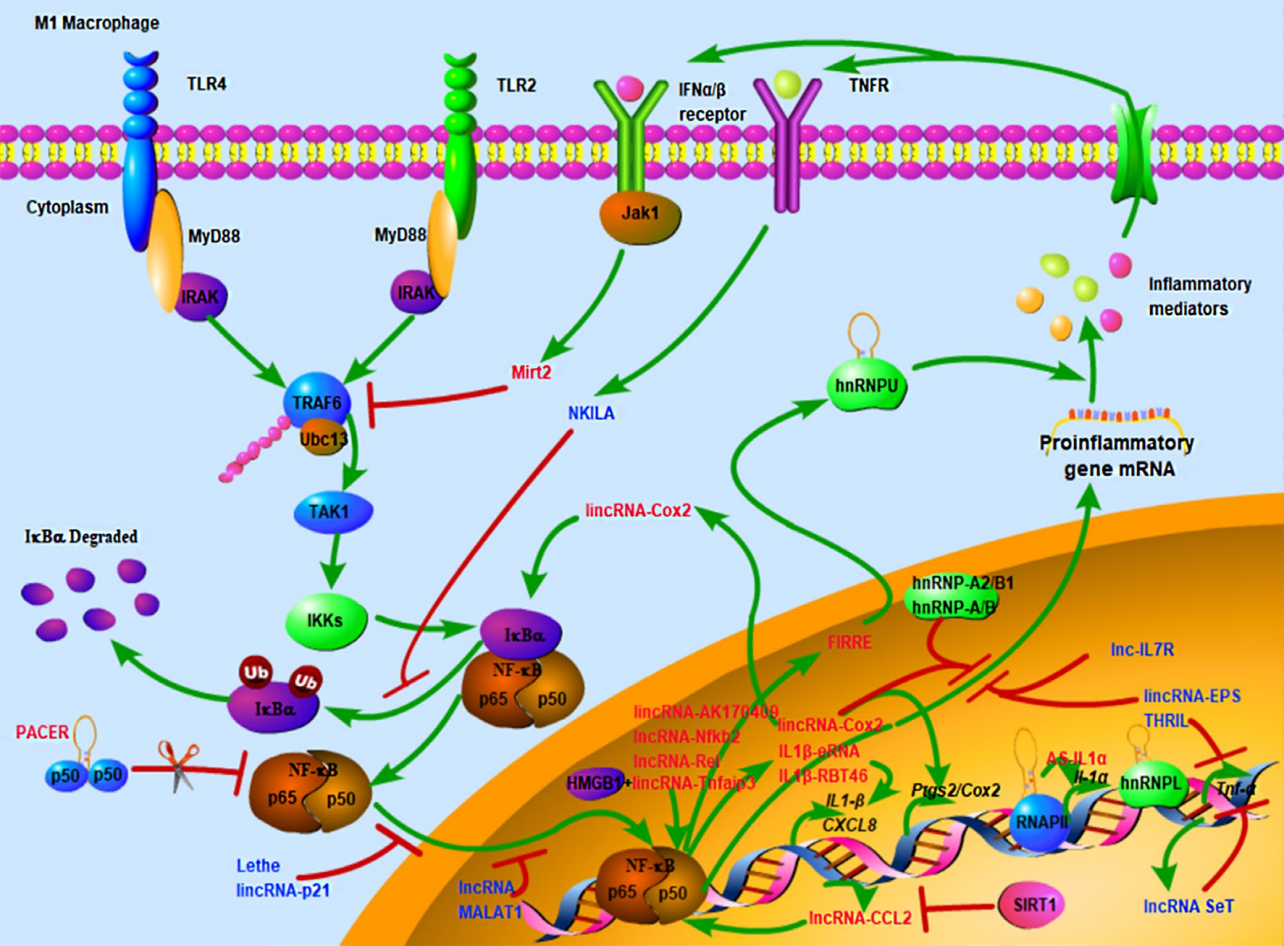
Involvement and regulation of lncRNAs in M1‐like macrophage polarization. Many toll‐like receptor (TLR)‐triggered polarization of M1‐like macrophage mainly depends on nuclear factor‐κB (NF‐κB) signalling pathway. At the plasma membrane, the binding of myeloid differentiation marker 88 (MyD88) to TLRs results in the recruitment and phosphorylation of IL‐1 receptor‐associated kinases (IRAKs), which facilitates oligomerization (via K63) and auto‐ubiquitination (via E2 ubiquitin ligase Ubc13) of TNF receptor‐associated factor 6 (TRAF6). Ubiquitinated TRAF6 subsequently activates other signalling proteins, such as transforming growth factor β‐activated kinase (TAK1). Then TAK1 activates the inhibitor of κB α (IκBα) kinases (IKKs), leading to degradation of IκBα and activation of NF‐κB, immune and inflammatory responses. LncRNAs promote the development of M1 macrophage via NF‐κB or other pathways showed in red font, whereas negative regulated displayed in blue font^50^, ^51^, ^52^, ^53^

Granulocyte macrophage colony stimulating factor (GM‐CSF), LPS and Toll‐like receptor (TLR) ligands always contribute to M1 polarization in macrophage. That macrophage might be polarized through the JNK pathway after binding to CCR2 and nuclear factor‐κB (NF‐κB), or through the activation of PI3K with increased RelA/NF‐κB activity.^8^ Functionally, M1‐type macrophage promotes Th1 response and produces copious amounts of pro‐inflammatory cytokines or reactive oxygen species (ROS) to kill pathogens.^44^ TLR‐triggered NF‐κB is one of the best studied pathways participated in the conversion of macrophage to M1‐like phenotype,^49^ which is shown in Figure 3.^50^, ^51^, ^52^, ^53^ All TLRs, excluding TLR3, mainly activate the NF‐κB‐dependent or IRF7‐dependent type I IFN (TLR7‐9) pro‐inflammatory signallings via the adapter protein myeloid differentiation marker 88 (MyD88).^54^, ^55^ Many transcriptome sequencing analyses of macrophages stimulated with TLR ligands have shown that numerous genes and lncRNAs involved in macrophage polarization (Figure 3 and Table 1), and these macrophages tended to be induced into M1 type, along with high levels of pro‐inflammatory mediators.^56^

In response to TLR4 (LPS) activation, lncRNA‐Nfkb2 and lncRNA‐Rel, located near some classical pro‐inflammatory transcription factors, were up‐regulated and co‐regulated with inflammatory response in mouse bone marrow‐derived macrophages (BMDMs).^57^ AS‐IL1α was a NAT located in the nucleus of macrophage and expressed at a low level in resting cells. It was highly up‐regulated after stimulating by TLRs via NF‐ĸB signal, and then recruited RNPII to the *IL‐1α* promoter. In the presence of abundant AS‐IL1α, elevated acetylation of histone H3 at lysine 9 (H3K9) promoted the combination of RNPII and the promoter of *IL‐1α*, but it could not control the gene's expression*.* 58 LncRNA cox‐2 (also has been called PACER) was located at upstream of the *cyclooxygenase‐2* (*cox‐2*) gene in both human and murine macrophages. PACER could directly interact with the p50/p50 homodimeric, an inhibitor of NF‐κB family and subsequently resulted in activation of the *cox‐2* promoter.^49^, ^59^ Moreover, lnc IL1β‐eRNA and IL1β‐RBT46 were found to be transcribed from enhancer regions via NF‐ĸB in THP‐1 cells, and predominantly localized to the nucleus, functioning as eRNAs to promote the expression of IL1‐β and CXCL8.^60^ After LPS stimulation, lincRNA‐Tnfaip3, which is located at the tumour necrosis factor a‐induced protein 3 (*Tnfaip3*) gene, mediated early response through NF‐κB signalling in murine macrophages. LincRNA‐Tnfaip3 could non‐covalently unite to the high‐mobility group box 1 protein (HMGB1), DNA and RNA molecules, then assembled into a complex to facilitate *Hmgb1*‐associated histone modification and the combination of DNA and p65/p50, eventually promoted the transcription of many inflammatory and defence genes in macrophages,^61^ as well as other mice tissues induced by LPS.^62^ Sirtuin‐1 (SIRT1) is a conserved histone deacetylase, which can inhibit macrophage response by deacetylating H3K9 in lncRNA‐CCL2 loci and transcriptionally suppressing the lncRNA‐CCL2 level.^63^ LncRNA‐CCL2 was significantly up‐regulated in sepsis mice or LPS‐stimulated macrophages, but the SIRT1 expression was down‐regulated.^64^ Lu et al have reported that increased binding of p65 to the promoter of functional intergenic repeating RNA element (*FIRRE*) gene mediated lncRNA FIRRE up‐regulation in macrophages and intestinal epithelial cells after LPS stimulation. FIRRE was a NF‐κB‐controlled lncRNA and specifically interacted with hnRNPU, then regulated the mRNA stability of several genes by binding to the AU‐rich elements of these mRNA.^65^ LncRNA SNHG16 was released into cell cytoplasm and could act as a ceRNA of miR‐15a/16 to up‐regulate TLR4 in RAW264.7 cells.^66^

TLR2 activation could induce a high lincRNA‐Cox2 level and promote its neighbouring prostaglandin‐endoperoxide synthase 2 (*Ptgs2/Cox2*) gene expression in BMDMs through MyD88/NF‐κB signalling pathway, mediating an early inflammatory gene production.^67^ After lincRNA‐Cox2 being recruited to the SWItch/Sucrose Non‐Fermentable (SWI/SNF) complex in macrophages, NF‐κB subunits subsequently bound to this complex, finally triggered SWI/SNF‐associated chromatin remodelling and transcription of late inflammatory genes.^68^ A RNA‐seq analysis in TLR‐activated BMDMs indicated that lincRNA‐Cox2 and lincRNA‐AK170409 were involved in controlling NF‐κB signalling. Unlike lincRNA‐Cox2 causing IκBα degradation in cell cytoplasm, lincRNA‐AK170409 was only retained in the nucleus and up‐regulated various inflammation genes by affecting other components of NF‐κB.^69^ Meanwhile, lincRNA‐Cox2 could act as a suppressor of inflammation by directly interacting with the hnRNP‐A2/B1 and hnRNP‐A/B.^67^ Additionally, in intestinal epithelial inflammatory, lincRNA‐Cox2 significantly decreased the transcription of *Il12b* (a late‐responsive cytokine) by mobilizing the Mi‐2/nucleosome remodelling and deacetylase (Mi‐2/NuRD) repressor complex to the promoter region of *Il12b*, which led to increased H3K27 dimethylation at the promoter and suppressive expression of *Il12b*.^70^

LncRNA Lethe, lincRNA‐p21 and lnc‐IL7R were increased in macrophages activated through NF‐κB pathway, and acted in a negative feedback loop to inhibit NF‐κB‐induced inflammation. Mechanically, lncRNA Lethe combined with the p65 subunit of NF‐κB and kept it away from DNA, and then presented an anti‐inflammatory effect by blocking the activation of downstream inflammatory genes, NADPH oxidase 2 (NOX2) expression and ROS production.^71^ LincRNA‐p21 was also found to attenuate the p65 mRNA and its translation, leading to TNF‐α‐stimulated NF‐ĸB activity.^72^ Increased lnc‐IL7R was initially expressed in the nucleus and likely inhibited MyD88‐dependent LPS‐inducible genes expression by enhancing the trimethylation of H3K27, a mark of transcriptional silence, at its promoters. However, it did not seem to involve in regulating the expression of overlapped *IL7R* gene and viral infection.^73^ Similarly, lincRNA THRIL as a negative regulation of NF‐κB could interact with hnRNPL at the *Tnfα* gene and ensure the expression of TNF‐α in dosage control, but it down‐regulated in human macrophages after TLR2 activation.^74^, ^75^ Encoded by the *Tnfα* loci, lncRNA SeT increased in LPS‐treated murine macrophage,^76^ with unstable *Tnfα* mRNA.^20^ The above lncRNAs are important to reduce TNFα expression in macrophages. NKILA was highly expressed to restrain the NF‐ĸB‐driven inflammation in response to TNF‐α irritant. NKILA could decorate the phosphorylation of IĸBα to inhibit its degradation and prevent NF‐ĸB from being transferred to the nucleus.^77^ LincRNA‐EPS in resting macrophages, which inversely controls the level of IL‐6, was overexpressed but reduced via TLR ligation triggered NF‐ĸB pathway. Atianand et al^78^ revealed that the recruitment of RNPII and trimethylation of H3K4 near the transcription start site (TSS) of immune response genes were transcriptionally suppressed by lincRNA‐EPS, which functioned as a scaffold to hnRNPL. LncRNA MALAT1 was up‐regulated in LPS‐activated macrophage. In the nucleus, it can combine with NF‐κB and keep its complex from binding DNA, then inhibit the expression of inflammatory cytokines.^79^ Knocking down the MALAT1 in LPS‐induced murine alveolar macrophage MH‐S showed a lower inflammatory response by up‐regulating the miR‐146a expression.^80^ LincRNA MacORIS was located in cell cytoplasm, and served as a depressor of IFN‐γ in THP‐1 macrophage, leading to decrease in IFN‐γ–induced Janus kinase 2 (JAK2) and phosphorylation of signal transducer and activator of transcription 1 (STAT1), but that did not happen in mouse macrophages.^81^

### 4.2 Characteristics of lncRNAs in M2‐like macrophage, M1/M2 switch or TMAs

Macrophage activated through an opposite manner is known as M2 type. M2 macrophage plays an essential role in adaptive response and contributes to Th2‐type response involved in acute tissue damage and repair.^44^, ^47^ However, studies on the expression of lncRNAs in M2 macrophage are still rare, and the exact mechanism of action is still unclear. Du et al found that lncRNA Mirt2 was up‐regulated in the cytoplasm of macrophage after LPS stimulating, then inhibited the oligomerization and ubiquitination of TNF receptor‐associated factor 6 (TRAF6) and relieved inflammatory responses at the late stage. They speculated that Mirt2 might hide the ubiquitination sites for the E2 ubiquitin ligase Ubc13, and induce a decreased ubiquitination level of TRAF6 (Figure 2 and Table 1). At the early stage, IL‐4 could reduce Mirt2 via the Jak‐Stat6 pathway. After restoring the expression of Mirt2, IL‐4 treated macrophage was inclined to M2 polarization with a high level of M2 molecule markers. However, the elaborate mechanisms of macrophage polarization induced by Mirt2 are still unknown.^53^ Murphy et al conducted real‐time PCR in LPS‐tolerized macrophage and found increased expression of lncRNA HAR1A and HAR1B but decreased levels of PCGEM1 and HOTTIP lncRNAs.^50^ Regulating the expression of HOTTIP is associated with TLR tolerance, and has been speculated to promote macrophage into M2 phenotype.^82^ In polymethyl methacrylate (PMMA)‐induced BMDMs, the expression of TNF‐α, iNOS and miR‐21a‐5p were increased, but the expression of IL‐10, Arg1 and lncRNA KCNQ1OT1 were suppressed. KCNQ1OT1 might act as a miR‐21a‐5p decoy to up‐regulate IL‐10 expression,^83^ and then prompt macrophages into M2 type.^84^ A detailed transcriptome analysis in human macrophage revealed that lncRNA TCONS_00019715 was highly expressed when M2 type was converted to M1, but decreased when M1 type was converted to M2. Like TCONS_00019715, overexpressed *P21‐activated kinase 1 (PAK1)* in macrophage was helpful to M1 macrophage polarization.^85^ During M2 polarization, lncRNA‐MM2P was increased, which was contrary to M1 macrophage. Knockdown of lncRNA‐MM2P in RAW264.7 could cut down the M2 polarization by decreasing phosphorylation level of STAT6.^86^ LncRNA growth‐arrest‐specific transcript 5 (GAS5) was up‐regulated in human monocyte‐derived macrophages from pneumonia children and acted as a miR‐455‐5p decoy to up‐regulate SOCS3, then inhibited JAK2/STAT3 signalling and increased the transformation of M1 macrophage^87^ (Figure 2 and Table 1).

Tumour‐associated macrophages (TAMs) have a particular polarization state which is deemed as M2‐like macrophage.^47^ Incubated in thyroid tumour microenvironment, macrophage showed an increased expression level of MALAT1, which mediated secretion of fibroblast growth factor‐2 (FGF2) protein, leading to decreased cytokine release, enhanced tumour neovascularization, proliferation of thyroid cancer cells and metastatic potential.^3^, ^88^ In TAMs isolated from thyroid cancer, expression of lncRNA nuclear enriched abundant transcript 1 (NEAT1), which is localized in the nucleus (24), and Arg‐1 were obviously elevated, while miR‐214 was significantly decreased. Indirectly, NEAT1 promoted thyroid cancer growth by inducing β‐catenin expression.^89^ TAM‐derived exosomal miR‐146b‐5p might target with *TRAF6* gene, and inhibit endothelial cells migration through decrease in matrix metalloproteinase‐2 (MMP2). After co‐culturing with epithelial ovarian cancer (EOC)‐derived exosomes, this inhibition was reversed remarkably. Additionally, lncRNA ENST00000444164 and ENST0000043768 were overexpressed in EOC‐derived exosome and might regain the migration of endothelial cell via NF‐κB phosphorylation.^90^ In hepatocellular carcinoma (HCC), lncRNA cox‐2 could suppress the tumorigenesis and metastasis by promoting M1‐type macrophage and inhibiting M2 type.^56^ LncRNA uc.306 was up‐regulated in U937 cells during M2 differentiating to M1 phenotype. Uc.306 was also low expressed in HCC tissues, indicated a potential prognostic biomarkers of HCC.^91^ Breast cancer cells showed a high expression of lncRNA urothelial cancer‐associated 1 (UCA1) when cultured with macrophages. UCA1 could promote the proliferation, migration and angiopoiesis of tumour cells, and had a good correlation with the progress of breast cancer.^92^ Chen et al proved that aerobic glycolytic tumour cells released lactate to up‐regulate the EV‐transmitted lncRNA HISLA in macrophages, which induced chemoresistance and shorter survival of patients with breast cancer by blocking the interaction of PHD2 and HIF‐1α and inhibiting the hydroxylation and degradation of HIF‐1α.^93^ When co‐cultured with prostate cancer cell line PC‐3 cells, miR‐148a was highly expressed in TAMs, whereas CCAT1 and PKCζ were highest in M1 macrophage. Down‐regulated lncRNA CCAT1 promoted M2 macrophage polarization by up‐regulating miR‐148a, and then down‐regulating the expression of PKCζ.^94^

In addition to M1, M2 type and TAMs, some macrophage subsets with unique characteristics and functions have also been reported, such as CD169+ or TCR+ macrophage.^47^ However, it is still not clear about these newly discovered macrophage types, and more research is needed to prove its origin, biomarkers and differentiation processes. Hence, we only reviewed classical M1, M2 macrophage and TAMs.

## 5 INFLAMMATORY DISEASES INDUCED BY DYSREGULATION OF LNCRNAS AND MACROPHAGES

### 5.1 Atherosclerosis and cholesterol transport

Macrophages take up oxidized low‐density lipoproteins (oxLDL), causing apoptosis to form foam cells, which is the basis of atherosclerosis.^95^ A transmembrane protein, the type B scavenger receptor CD36, is up‐regulated on macrophage's cell membrane by oxLDL uptaking during this progress.^96^ After oxLDL treatment, lncRNA MALAT1 transcription was activated through NF‐κB signal. Numerous expressed lncRNA MALAT1 promoted CD36 expression through recruiting β‐catenin to the promoter region of CD36, and participated in CD36‐mediated lipid uptaking.^97^, ^98^ LncRNA MALAT1 expressed in macrophage of diabetic atherosclerosis (DA) was also obviously increased, which might promote the pyroptosis of normal macrophage. Low dose of chronic sinapic acid (SA) could down‐regulate lncRNA MALAT1 in DA rats and release the pyroptosis.^99^ When THP‐1 cells treated with oxLDL, the expression of lncRNA HOTAIR was significantly elevated, followed by decrease in miR‐330‐5p, which mediate low oxidative stress and inflammation.^100^ After binding to the E3 ubiquitin‐protein ligase and mouse double minute 2 (MDM2), lincRNA‐p21 could heighten the transcription of p53 by forcing MDM2 released from the p53 (cell cycle and apoptosis control molecule), subsequently allowed p53 binding to p300 and acted on their target genes. But in atherosclerotic plaques, expression of lincRNA‐p21 was depressed.^101^ LncRNA taurine up‐regulated gene 1 (TUG1) was elevated in HFD‐treated ApoE‐/‐ mice, oxLDL‐induced mouse macrophages and mouse VSMCs. As a target of TUG1, miR‐133a was correspondingly reduced and reversed the expression of fibroblast growth factor 1 (FGF1) protein, further inhibited cell apoptosis and aggravated the foam cell formation.^102^ Another increased lncRNA H19 in oxLDL‐treated macrophage might facilitate effects on foam cell formation and inflammation response by reducing miR‐130b.^103^ Hu et al revealed that in THP‐1‐derived foam cells, lncRNA RP5‐833A20.1 (NFIA‐A1) was increased but the transcription factor nuclear factor 1A (*NFIA*) was decreased. Besides, induced high miR‐382‐5p level might diminish the NFIA mRNA and protein.^104^ Further reports elucidated that *NFIA* could promote retrograde cholesterol transport (RCT), reduce the circulating levels of pro‐inflammatory cytokines and significantly improve plasma hyperlipidaemia.^105^ Transmembrane protein ATP‐binding cassette transporter A1 (ABCA1) takes part in the process of RCT. Human high‐density lipoprotein (HDL) deficiencies, such as syndrome sterol deposits and premature atherosclerosis, are caused by ABCA1 mutations.^106^ In THP‐1 derived foam cells, lincRNA‐DYNLRB2‐2 was distinctly increased, which promoted ABCA1‐mediated RCT and inhibited inflammation response by up‐regulating G‐protein‐coupled receptor (GPR119), and eventually slowed the atherosclerotic plaque formation down.^107^ Sallam et al have identified the lncRNA MeXis positive regulated the critical cholesterol efflux gene *Abca1,* which encodes ABCA1 protein. Lack of MeXis in mouse BMDMs affected the structure of the *Abca1* loci, had a negative impact on cholesterol overload and promoted plaque formation. Mechanically, the transcriptional coactivator DDX17 was recruited to the promoter of MeXis and magnified the LXR‐dependent transcription of *Abca1*.^108^ Work by Chen et al^109^ showed that lncRNA GAS5 was highly expressed not only in the plaque of atherosclerosis patients but also in animal models. Some reports indicated that the elevated lncRNA GAS5 might motivate the apoptosis of THP‐1‐derived foam cells,^110^ whereas other study confirmed that the enriched GAS5 could exacerbate the secretion of pro‐inflammatory cytokines and chemokine induced by oxLDL. Additionally, GAS5 suppressed the miR‐221 expression as a sponge, which might cause plaque destabilization.^111^ After oxLDL treatment, lncRNA GAS5 was abundant in THP‐1‐derived exosomes and enhanced vascular endothelial cell apoptosis by sucking up the exosomes.^112^

### 5.2 Diabetes mellitus

Transcriptome profiling of BMDMs from diabetic db/db mice indicated a pro‐inflammatory, pro‐fibrogenic and disordered polarization of macrophage. A RNA sequencing and real‐time qPCR demonstrated that lncRNA Dnm3os (dynamin 3 opposite strand) was up‐regulated via NF‐κB activation in BMDMs from type 2 diabetic db/db mice as well as monocytes from type 2 diabetic patients. Stable overexpression of Dnm3os in macrophages altered global histone modifications and promoted inflammation, immune response and phagocytosis.^113^ LncRNAs E330013P06 (E33) is one of the visibly increased lncRNAs in macrophages under T2DM conditions. Compared with db/+ mice, pro‐inflammatory genes were overexpressed from db/db mice, which indicated that diabetes inhibited M2 phenotypic transformation and promoted the inflammatory response of M1 macrophage. Additionally, expression of E33 in macrophages greatly increased the CD36 level, and further supported the foam cell formation and proatherogenic responses of macrophages.^114^ A new study indicated that, under high glucose conditions or diabetic wounds, lncRNA Lethe was decreased in macrophages, and released more free p65‐NF‐κB to the nucleus, which promoted ROS and NOX2 production, eventually impaired wound healing.^115^

### 5.3 Coeliac disease

Coeliac disease (CeD) is a immune‐mediated primary intestinal malabsorption syndrome caused by gluten intolerance, and the pathological feature is small intestinal mucosal lesions.^116^ LncRNA lnc13 contains a CeD‐associated haplotype block and expresses at a low level in resting cells. A low level of lnc13 was detected in CeD patients’ small intestinal biopsy samples and BMDMs treated with LPS stimulus. The overall expression of decapping protein 2 (Dcp2) was inversely increased in the above specimens. It showed that increased Dcp2 could promote the degradation of lnc13 by separating the lnc13‐hnRNPD compound and relieve subsequent inflammatory respond after LPS stimulating.^117^ In the cytoplasm of human coeliac disease patient samples and macrophages, the functional characteristics of increased cardiac and apoptosis‐related lncRNA (Carlr) was defined, which elevated the expression of NF‐κB‐regulated genes through binding to p65 and letting it released from IκBα. Aberrant activation of NF‐κB might cause the overproduction of inflammatory cytokines and mucosal inflammation that contributes to intestinal inflammatory diseases. At the same time, NF‐κB signalling caused changes in other lncRNAs that have been shown to be important for both cytokines production and subsequent resolution of inflammation. Compared with control patients, three of the putative Carlr‐regulated targets, TNFAIP3, IL1B and PTGS2, showed statistically significant higher expression in coeliac patients.^118^

### 5.4 Mycobacterial infections

Macrophages can phagocytose invading mycobacterium tuberculosis (Mtb), so it is considered as the main residence for Mtb and gives the ability to escape damage. LncRNAs have proved to involve in anti‐mycobacterial infection, but their effects on this response remain unelucidated.^119^ An arrayed analysis of lncRNAs was conducted in human macrophages infected with M. bovis Calmette‐Guerin (BCG), and showed that several lncRNAs expression including MEG3 was suppressed after infection. MEG3 was associated with mTOR and PI3K/AKT pathway and involved in autophagy regulation of macrophages using pathway analysis. And persistent decline in MEG3 expression was observed in IFN‐γ‐induced autophagy of infected macrophages. Knockdown of MEG3 in macrophages would help to destroy the intracellular M. bovis BCG of macrophages.^120^ Another microarray analysis conducted in human macrophages with H37Ra or H37Rv about 72 hours, revealed that lncRNAs diversity expressed in macrophages. In particular, up‐regulated MIR3945HG V1 and MIR3945HG V2 showed potential diagnostic markers for Mtb infection, but their functions were still unknown.^121^

### 5.5 Other relevant diseases

**Figure 4:**
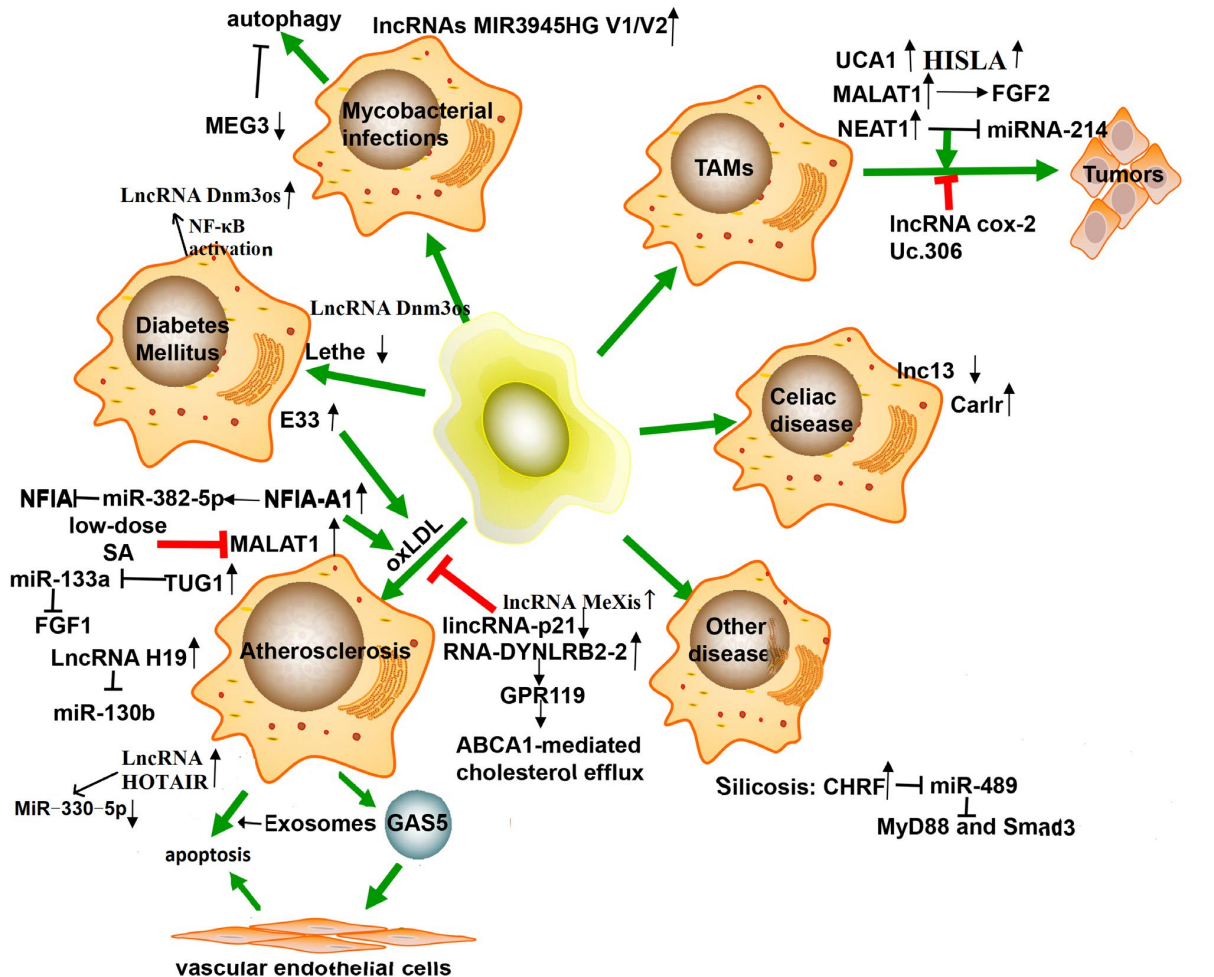
Dysregulation of lncRNAs and macrophages in inflammatory diseases. Macrophages are closely related to a number of physiological courses and pathological changes. Major lncRNAs involved in each macrophage‐mediated key processes have been presented. TAMs: tumour‐associated macrophages

Silicosis is one of the most severe occupational diseases, and shows common histological changes in persistent inflammation and pulmonary fibrosis. Studies have indicated that silica particles could promote the liberation of copious oxidants and inflammatory factors in macrophages and epithelial cells, which caused a deposition in extracellular matrix, and finally led to pulmonary fibrosis.^122^ Silica‐induced pulmonary fibrosis, macrophages exposed to silica or fibroblasts exposed to TGF‐β were decreased the miR‐489 level.^123^ MiR‐489 possesses the anti‐fibrosis action via decreasing MyD88 and Smad3 target. LncRNA cardiac hypertrophy‐related factor (CHRF) was elevated and accelerated inflammation responses and fibrosis in the silica‐treated RAW264.7, then acted as a sponge of miR‐489, reversing the inhibitory effect of miR‐489^124^ (Figure 4).

## 6 CONCLUSION

Macrophages are remarkably versatile, and can elicite extensive responses to environmental stimulus while maintaining organ homeostasis and preventing autoimmunity. LncRNAs have been demonstrated to participate in multiple stages of immune cell development and in pathogen responses. Combining currently published articles, we witness an significant growth in the understanding of lncRNAs regulation on macrophage biology, including its development, differentiation and activation. Such understandings of how lncRNAs function in macrophages may influence future therapies for inflammation‐associated diseases, and add a new perspective for the innate inflammatory response. The same lncRNA expressed in macrophages can play diversities of roles in different physiological and pathological processes, such as lncRNA MALAT1. Some lncRNAs, however, both inhibit and promote inflammation reponses through different mechanisms in macrophages in the case of internal environment disorder, like linc RNA‐Cox2. Besides, this review summarizes the interaction between lncRNAs and some miRNAs in the process of macrophage development. Exosomal‐derived lncRNAs also are involved in diverse biological progress.^125^ Macrophage‐secreted exosomes can transport lncRNAs, regulate and modify its development and polarization, indicating its remote regulation in life processes, and adding a new dimension to macrophage functions. Circular RNAs (circRNAs) are new molecules in the regulation of post‐transcriptional genes expression and important to the pathological process of several diseases. According to previous researches, circRNAs play a crucial role in fine‐tuning the level of miRNA‐mediated regulation of gene expression by sequestering miRNAs. However, the effects of circRNAs in macrophage differentiation and polarization have not to be widely explored. Whether circRNA involved in the activation of macrophages and whether it affect the development of macrophages by regulating lncRNAs require more further experiments to detect.^126^ Unfortunately, there is a defect in our paper that many studies on the mechanisms of lncRNAs expressed in macrophages have not been studied intensively. It is expected that more exploration about lncRNAs in regulating innate immune response and macrophage‐associated biological processes will be carried out. In conclusion, lncRNAs harmonizes in the development and function of macrophages, which can prevent the human body from being damaged by internal and external stimuli without generating an excessive immune response. Increased or depressed lncRNAs in macrophages might be regarded as potential therapeutic targets and diagnostic biomarkers, and provided a new sight into the treatment of inflammatory diseases and cancers.

## CONFLICT OF INTEREST

The authors confirm that there are no conflicts of interest.

## AUTHORS CONTRIBUTION

Yixn Xie and Min Wang initiated this review, read lots of literature and wrote the manuscript. Other authors revised our first draft and provided valuable comments. All authors read the manuscript and approved it.
